# Effects of L-Citrulline Supplementation on Endothelial Function, Arterial Stiffness, and Blood Glucose Level in the Fasted and Acute Hyperglycemic States in Middle-Aged and Older Adults with Type 2 Diabetes

**DOI:** 10.3390/nu17233739

**Published:** 2025-11-28

**Authors:** Yejin Kang, Katherine N. Dillon, Danielle E. Levitt, Arturo Figueroa

**Affiliations:** 1 Department of Kinesiology and Sport Management, Texas Tech University, Lubbock, TX 79409, USA; yejin.kang@ubc.ca (Y.K.); kdillon@albion.edu (K.N.D.); danielle.levitt@ttu.edu (D.E.L.); 2 Department of Anesthesiology, Pharmacology & Therapeutics, University of British Columbia (UBC), Vancouver, BC V6T 1Z3, Canada; 3 UBC Centre for Heart Lung Innovation, St. Paul’s Hospital, Vancouver, BC V6Z 1Y6, Canada; 4 Department of Kinesiology, Albion College, Albion, MI 49224, USA

**Keywords:** hyperglycemia, arterial stiffness, blood glucose, endothelial function, L-citrulline supplementation, Type 2 diabetes mellitus

## Abstract

**Background:** Acute and chronic hyperglycemia in patients with type 2 diabetes mellitus (T2DM) causes endothelial dysfunction and arterial stiffness, contributing to early mortality from cardiovascular disease (CVD). Although L-citrulline (CIT) supplementation improves endothelial function in older women and decreases fasting glucose in those with T2DM, whether it improves vascular function and blood glucose during acute hyperglycemic states in T2DM is unknown. **Methods:** We randomized 16 patients with T2DM (age 62 ± 6 years) to consume either CIT (6 g/day) or placebo for 4 weeks. Brachial artery flow-mediated dilation (FMD), brachial and aortic blood pressure, aortic and leg arterial stiffness (pulse wave velocity, PWV), and blood glucose concentration were assessed in the fasted state and 60 min following glucose ingestion (during acute hyperglycemia). **Results:** Four weeks of L-citrulline supplementation improved FMD, femoral-ankle PWV, aortic systolic blood pressure, and blood glucose concentration in the fasted state compared to placebo (all *p* < 0.05). During acute hyperglycemia, CIT supplementation increased FMD and reduced femoral-ankle PWV, aortic systolic BP, and glucose levels compared to placebo (all *p* < 0.05). **Conclusions:** CIT supplementation is beneficial to improve vascular function and glucose levels during chronic and acute hyperglycemia in middle-aged and older adults with T2DM.

## 1. Introduction

Type 2 diabetes mellitus (T2DM) is recognized as a major metabolic disease with a rapidly increasing global prevalence. T2DM is characterized by a chronic state of hyperglycemia resulting from insulin resistance [1]. Insulin stimulates endothelial nitric oxide synthase (eNOS) activity and increase nitric oxide (NO) production, leading to vasodilation [2]. Endothelial insulin resistance promotes the secretion of endothelin-1, a vasoconstrictor, and diminishes NO production, a potent mediator of vascular health, causing endothelial dysfunction in T2DM [2]. Chronic hyperglycemia in T2DM leads to endothelial dysfunction and vascular complications through several mechanisms including increased oxidative stress, reduced NO bioavailability, and accumulation of advanced glycation end products [2,3]. Hyperglycemia-induced endothelial dysfunction contributes to the development of hypertension and cardiovascular disease (CVD) [4]. Aging and T2DM are closely associated with increased arterial stiffness and hypertension [5], which are consequences of endothelial dysfunction and independent predictors of CVD [6].

Acute postprandial hyperglycemia serve as a stronger indicator of future CVD mortality than fasting blood glucose in people with T2DM [7]. Oral glucose tolerance test (OGTT) is used to assess glycemic control, identify individuals with impaired glucose tolerance or T2DM, and estimate insulin resistance [8]. In normal individuals, blood glucose peaks mostly within 30–60 min of OGTT, whereas patients with T2DM exhibit elevated blood glucose levels for longer than 120 min [9,10,11]. Acute hyperglycemia-related oxidative stress caused by oral glucose ingestion leads to reduced endothelial-mediated vasodilation [10] and increased arterial stiffness and systolic blood pressure (BP) [12]. Indeed, previous studies have reported increased peripheral systolic BP (SBP) and arterial stiffness in men with T2DM after a standard breakfast [13]. Moreover, patients with T2DM experience the greatest decrease in FMD during OGTT at 60 min compared to controls [9,10], coinciding with the glucose peak at 60 min of OGTT [9].

L-arginine (ARG) is the only substrate for NO production and a key regulator of endothelial-mediated vasodilation [14,15]. Reduced ARG availability to eNOS is a main mechanism for endothelial dysfunction in aging and T2DM [16,17]. Increased oxidative stress up-regulates arginase activity [18], an enzyme competing with eNOS for their common substrate, ARG. Although evidence has shown improvement in endothelial function after short-term ARG supplementation [14], long-term ARG administration becomes ineffective due to increased ARG catabolism [18]. L-citrulline (CIT) is an effective precursor of ARG that indirectly enhances NO synthesis. Since oral CIT is not catabolized by arginase or extracted by the liver, CIT intake increases more circulating ARG concentration than a similar dose of ARG [15]. In people with T2DM, CIT supplementation has improved NO bioavailability, fasting glucose, and antioxidant capacity as well as inhibiting arginase activity [17,19,20,21]. In addition, CIT supplementation has increased brachial flow-mediated dilation (FMD), an assessment of endothelial function, in postmenopausal women with hypertension [22] and patients with heart failure [23]. Moreover, previous studies showed reductions in systemic and peripheral arterial stiffness, but not aortic stiffness, following CIT supplementation in middle-aged adults [24,25]. A study that examined the effect of 2 weeks of watermelon, which is rich in CIT, during acute hyperglycemia observed increased postprandial FMD area under the curve, but no changes in FMD% in healthy young adults [26]. However, the impact of dietary supplementations on vascular function during acute hyperglycemia in T2DM is still underexplored. Thus, the purpose of this study was to investigate the effect of 4-week CIT supplementation on endothelial function, arterial stiffness, aortic BP, and blood glucose levels in the fasted state and during acute hyperglycemia in patients with T2DM. We hypothesized that CIT supplementation would improve endothelial function, peripheral arterial stiffness, SBP, and blood glucose levels in the fasted state and attenuate the exacerbation of those impairments during acute hyperglycemia.

## 2. Materials and Methods

### 2.1. Subjects

Patients with T2DM (aged 50–75 years old) were enrolled from the general community of Lubbock, Texas via flyers and online advertisements between May 2023 and March 2024. Participants were included if they were diagnosed with T2DM by a physician at least 3 months prior to the beginning of this study; treated with oral hypoglycemic medications and/or insulin injections (Supplementary Table S1); had resting SBP < 160 mmHg and body mass index (BMI) < 40 kg/m^2^. All women had absence of menstruation for at least 1 year. If they had type 1 diabetes, uncontrolled T2DM (fasting blood glucose > 200 mg/dL) or any severe cardiovascular, renal, or pulmonary diseases, they were excluded from the study. Participants were also excluded if they reported smoking, greater than moderate consumption of alcoholic drinks (>7 standard drinks per week for women and >14 per week for men), consuming arginine- or CIT-rich foods and/or supplements, or if they were involved in structured, moderate or high intensity (>120 min/week) of exercise or physical activity within the last 6 months.

### 2.2. Experimental Design and Protocol

This study was a double-blind, randomized, placebo-controlled, crossover design. Randomization was performed by the principal investigator (AF), who was not involved in laboratory measurements using a block scheme stratified by age, SBP, and fasting blood glucose levels with a computer program. Using a block size of 4, 22 participants were randomly assigned in a 1:1 to CIT or placebo groups for 4 weeks and they were crossover to the opposite treatment after 8 weeks of washout. Blinding was maintained for participants and researchers involved in laboratory measurements. The allocation codes were kept in a sealed file and remained concealed until the unblinding phase. This study was approved by the Texas Tech University Institutional Review Board (IRB2022-1056; approved on 21 November 2022) and registered in ClinicalTrials.gov under NCT06016478 (registered in May 2023). Pre-qualified participants via phone-call screening visited the Vascular Health Laboratory at Texas Tech University to determine if they meet the qualifications for this study. All laboratory measurements were performed in the morning between 7–10 AM after an overnight fast (~8 h). Participants were also asked to abstain from medications, supplementations, and caffeine (~12 h) and alcohol consumption (~24 h).

On visit 1 (screening visit), the study protocols were fully explained to participants. Thereafter, they signed an informed consent and completed a health and exercise history questionnaire. Fasting blood glucose and hemoglobin A1C (HbA1C) levels were obtained twice by finger prick and averaged. BMI was calculated as body weight (kg) divided by height squared (m^2^). After 20 min of rest in the supine position, brachial BP was taken at least twice using an automated oscillometric device (HEM-907XL; Omron Healthcare, Vernon Hill, IL, USA) and averaged with a <5 mmHg difference in SBP from two readings.

During experimental visits (visits 2–5), blood glucose and HbA1C levels were determined by finger pick, and blood sample was drawn via venipuncture. Following 20 min-rest in the supine position, brachial and aortic BP, arterial stiffness, and endothelial function were assessed in the fasted state. Thereafter, OGTT was performed to induce acute hyperglycemia. Participants consumed 75 g of glucose (Dextrose Powder; NOW Foods, Bloomingdale, IL, USA) dissolved in 235 mL of water within 5 min. As a glucose level at 1-h of OGTT is a stronger predictor than 2-h for diabetes and CVD risk [27], brachial and aortic BP, arterial stiffness, endothelial function, and glucose levels measurements were repeated at 60 min following glucose consumption (60 min-OGTT). After visit 2 (first pre-intervention) measurements, participants were randomly assigned to consume either 750 mg CIT (6 g/day) or placebo (Microcrystalline cellulose) (both from NOW Foods, Bloomingdale, IL, USA). They were instructed to ingest 4 capsules in the morning and evening, respectively, an hour before or after their meals, for 4 weeks (between visits 2 and 3). The rational for this prescription was based on previous work that observed improvements in brachial FMD%, aortic SBP, and faPWV [24,28]. The placebo and CIT were prepared in capsules with identical size, color, and shape. On visit 3, post-intervention assessments were performed in as same way as on visit 2. Following an 8-week washout period, participants completed visit 4 (second pre-intervention) measurements and received the opposite supplementation for an additional 4 weeks (between visits 4 and 5). After 4-weeks of the second intervention, all post-intervention measurements were repeated on visit 5. To calculate compliance, we counted the number of unconsumed capsules that participants returned on their laboratory visits at the end of each 4-week period.

### 2.3. Measurements

The primary outcome was endothelial function, measured by brachial artery FMD. Secondary outcomes were BP, arterial stiffness, blood glucose, HbA1C and insulin. All primary and secondary outcomes were assessed at visits 2–5.

#### 2.3.1. Blood Pressure and Arterial Stiffness

Brachial and aortic BP were obtained from the brachial artery using the SphygmoCor XCEL system (AtCor Medical, Sydney, Australia). Aortic pressure waveforms and BP were generated from the brachial waveforms via a generalized transfer function of the system’s software (SphygmoCor XCEL system, AtCor Medical, Sydney, Australia). Brachial and aortic SBP, diastolic BP (DBP), mean arterial pressure (MAP), and pulse pressure (PP) were determined by averaging at least two readings with a <5 mmHg difference in SBP.

Aortic (carotid to femoral pulse wave velocity, cfPWV) and leg (femoral to ankle, faPWV) arterial stiffness were measured using the SphygmoCor XCEL and CPV system (AtCor Medical, Sydney, Australia), respectively. The carotid-femoral pulse waveforms were acquired by applanation tonometry on the common carotid artery, and a sensor cuff placed on the proximal thigh, which inflated automatically to capture 10–15 consecutive pulse waves. Similarly, femoral-ankle pulse waveforms were captured by applanation tonometry on the common femoral and dorsalis pedis arteries. Each segment distance was measured using a metal segmometer. PWV were calculated as the segmental distance divided by the transit time. PWV measurements were averaged from at least two readings with a ≤0.3 m/s difference.

#### 2.3.2. Endothelial Function

Brachial artery FMD% was used to evaluate endothelial function using Doppler ultrasound with a linear-array 10-MHz transducer (LogiQ S7; GE Medical Systems, Milwaukee, WI, USA) as previously described [29]. Brachial FMD was calculated as: FMD (%) = (Peak diameter (mm) − Baseline diameter (mm))/Baseline diameter (mm) × 100. Shear rate was calculated as: Shear rate (sec^−1^) = 4 × Mean blood velocity (cm/s)/Diameter (cm). All measurements were performed by one researcher (Y.K.). The intraobserver variability (coefficient of variation) in 13 participants was 7.7%.

#### 2.3.3. Blood Samples

Fasting blood glucose (Contour Blood Glucose Monitor; Bayer, Leverkusen, Germany) and hemoglobin A1C (HbA1C) (A1CNow+; PTS Diagnostics, Whitestown, IN, USA) levels were measured by finger prick. Fasting blood glucose measure was performed twice and averaged from two readings, with intra-assay percent coefficient of variation (%CV) ≤ 1.9%. 10 mL of blood was drawn from an antecubital vein using a 21-gauge butterfly needle by a certificated phlebotomist. The blood samples were collected into serum separator tubes, then were centrifuged for 10 min at 1000 g, aliquoted, and stored in a −80 °C freezer for subsequent analysis. Insulin was assessed using an ELISA kit (80-INSHUE01.1, ALPCO Diagnostics, Salem, NH, USA), which has 100% cross-reactivity with human insulin. The intra-assay %CV was ≤ 3.3%, and the inter-assay %CV, calculated from the inter-assay control, was 3.5%. Homeostatic Model Assessment for Insulin Resistance (HOMA-IR) was calculated as: Fasting Glucose (mg/dL) × Fasting Insulin (µIU/mL)/405.

### 2.4. Statistical Analysis

An *a priori* power analysis was performed using G*Power (version 3.1.9.7, Dusseldorf, Germany) to determine sample size. Sample size was determined as 16 participants per groups based on a previous study that showed a significant improvement in brachial artery FMD by 1.4% after 4 weeks of CIT supplementation compared to placebo [22] (a power of 80%, α = 0.05). Normality of data was verified using the Shapiro-Wilk test. An independent *t*-test was used to detect potential differences between groups at baseline for all measurements. A two-way analysis of variance (ANOVA) with repeated measures with Bonferroni adjustments, where appropriate, was performed to determine changes in anthropometrics, HbA1C, fasting glucose, insulin, and HOMA-IR levels between groups (CIT and Placebo) over time (pre and post). A three-way ANOVA with repeated measures with Bonferroni adjustments was performed to determine changes in glucose, FMD, PWV, and BP between groups (CIT and Placebo), over conditions (Fasted and 60 min-OGTT), and over time (pre and post). If significant interactions were observed, pairwise comparisons with the Bonferroni adjustment were used as post-hoc tests. Changes in each variable (from fasted to 60 min-OGTT and/or from pre to post) were calculated and compared between groups using an independent *t*-test. Statistical analyses were performed using SPSS version 29.0 (IBM SPSS Inc., Chicago, IL, USA). Data was reported as mean ± SD in tables and mean ± SE in figures, and significance was set to *p* < 0.05.

## 3. Results

### 3.1. Participants

**Figure 1:**
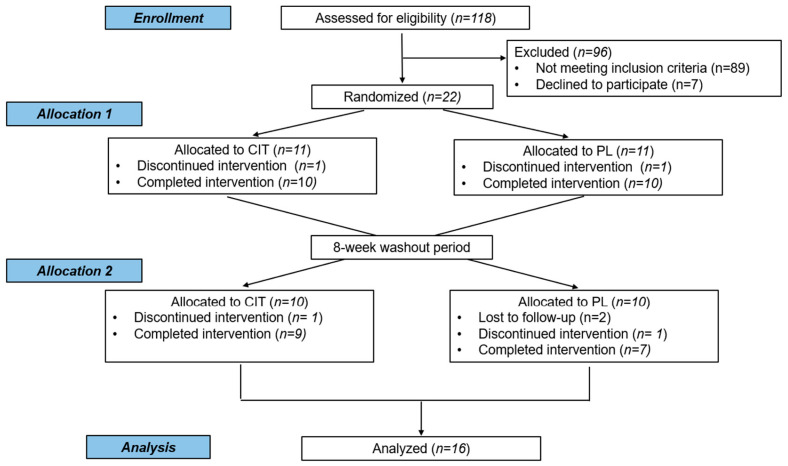
CONSORT flow of participants in the study. CIT, L-citrulline; PL, placebo intervention.

One hundred and eighteen individuals were assessed for participation eligibility, and 22 of eligible participants were randomly allocated to the first intervention of either CIT or placebo group. A total of 16 participants completed the crossover study and used for the final analysis (Figure 1). Participant characteristics and medications are reported in Table 1 and Table S1, respectively. Regarding compliance to supplementation, consumption of CIT and placebo was 92 ± 6% and 91 ± 7%, respectively. CIT supplementation is generally considered safe and well-tolerated for most individuals, but one participant reported that she experienced mild stomach discomfort.

### 3.2. Anthropometrics and Glucose Levels

There were no between group differences in any variable at baseline (all *p* < 0.05, Table 2). Significant group by time interactions were found for BMI (*p* < 0.05), waist circumference (*p* < 0.05), HbA1C (*p* < 0.01), glucose (*p* < 0.01), and insulin (*p* < 0.05). From pre to post, CIT supplementation decreased waist circumference (CIT: Δ−2.5 ± 3.4 cm vs. Placebo: Δ0.3 ± 2.8 cm, *p* < 0.05), HbA1C (CIT: Δ−3 ± 5 mmol/mol (Δ−0.3 ± 0.4%) vs. Placebo: Δ2 ± 5 mmol/mol (Δ0.2 ± 0.5%), *p* < 0.05), and glucose (CIT: Δ−14 ± 6 mg/dL vs. Placebo: Δ11 ± 5 mg/dL, *p* < 0.01) compared to placebo. The reduction in BMI was not statistically significant in CIT (*p* > 0.05, Table 2), but ΔBMI was different between groups (CIT: Δ−0.3 ± 0.7 kg/m^2^ vs. Placebo: Δ0.2 ± 0.5 kg/m^2^, *p* < 0.05). Importantly, although we did not observe statistical significance in HOMA-IR, there was a decreasing trend in CIT and an increasing trend in placebo after 4 weeks.

**Figure 2:**
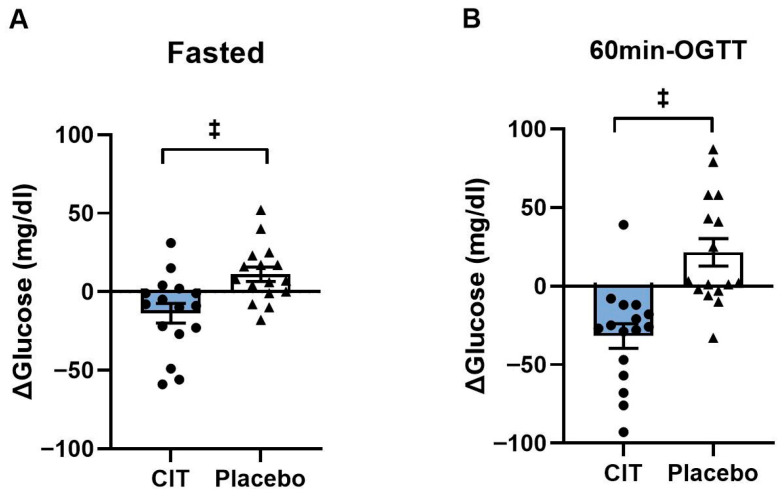
Changes (Δ) in blood glucose levels from pre to post (**A**) in the fasted state and (**B**) at 60 min of oral glucose tolerance test (60 min-OGTT) in L-Citrulline (CIT) and placebo groups. ^‡^ *p* < 0.01 vs. placebo.

Blood glucose levels were not different between groups before interventions (*p* > 0.05, Table 3). Significant group-by-time interactions were found both in the fasted state and at 60 min-OGTT (all *p* < 0.01, Table 3). Fasting blood glucose level was reduced by CIT compared to placebo (CIT: Δ−14 ± 6 mg/dL vs. Placebo: Δ11 ± 5 mg/dL, *p* < 0.01) (Figure 2A). In addition, CIT supplementation lowered blood glucose level at 60 min-OGTT compared to placebo (CIT: Δ−32 ± 3 mg/dL vs. Placebo: Δ22 ± 9 mg/dL, *p* < 0.01) (Figure 2B).

### 3.3. Endothelial Function

**Figure 3:**
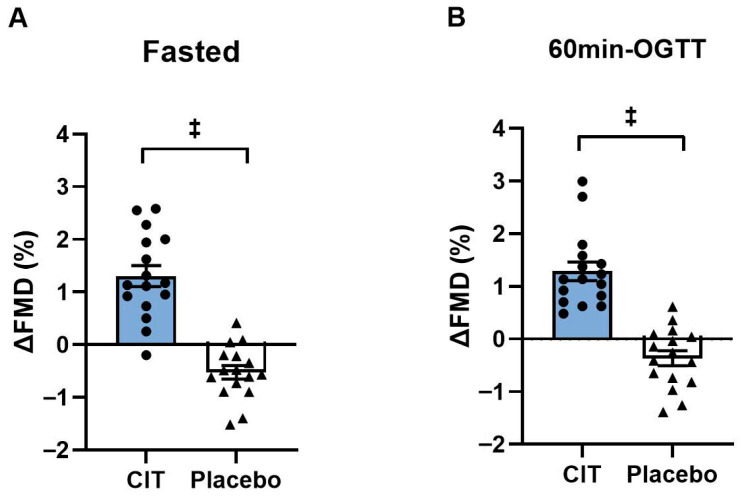
Changes (Δ) in brachial artery flow-mediated dilation (FMD) from pre to post (**A**) in the fasted state and (**B**) at 60 min of oral glucose tolerance test (60 min-OGTT) in L-Citrulline (CIT) and placebo groups. ^‡^ *p* < 0.01 vs. placebo.

At baseline, all brachial FMD variables did not differ between groups (all *p* > 0.05, Table 4). There were significant group-by-time interactions in FMD both in the fasted state and at 60 min-OGTT (all *p* < 0.01, Table 4). FMD improved in the fasted state after CIT supplementation compared to placebo (CIT: Δ1.3 ± 0.2% vs. Placebo: Δ−0.5 ± 0.1%, *p* < 0.01) (Figure 3A). Similarly, CIT enhanced FMD at 60 min-OGTT compared to placebo (CIT: Δ1.3 ± 0.2% vs. Placebo: Δ−0.4 ± 0.1%, *p* < 0.01) (Figure 3B).

### 3.4. Arterial Stiffness and Blood Pressure

**Figure 4:**
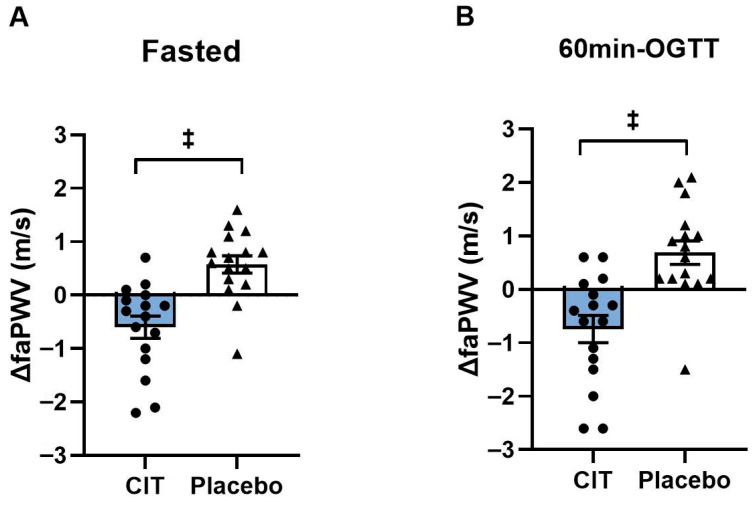
Changes (Δ) in femoral-ankle pulse wave velocity (faPWV) from pre to post (**A**) in the fasted state and (**B**) at 60 min of oral glucose tolerance test (60 min-OGTT) in L-Citrulline (CIT) and placebo groups. ^‡^ *p* < 0.01 vs. placebo.

At baseline, there were no differences in cfPWV and faPWV between groups (all *p* > 0.05). Significant group-by-time interactions were found in faPWV in the fasted and hyperglycemic states (*p* < 0.01, Table 5). Although there was no change in cfPWV, 4 weeks of CIT supplementation reduced faPWV in the fasted state (CIT: Δ−0.6 ± 0.2 m/s vs. Placebo: Δ0.6 ± 0.2 m/s, *p* < 0.01) and at 60 m-OGTT (CIT: Δ−0.7 ± 0.3 m/s vs. Placebo: Δ0.7 ± 0.2 m/s, *p* < 0.01) compared to placebo (Figure 4).

**Figure 5:**
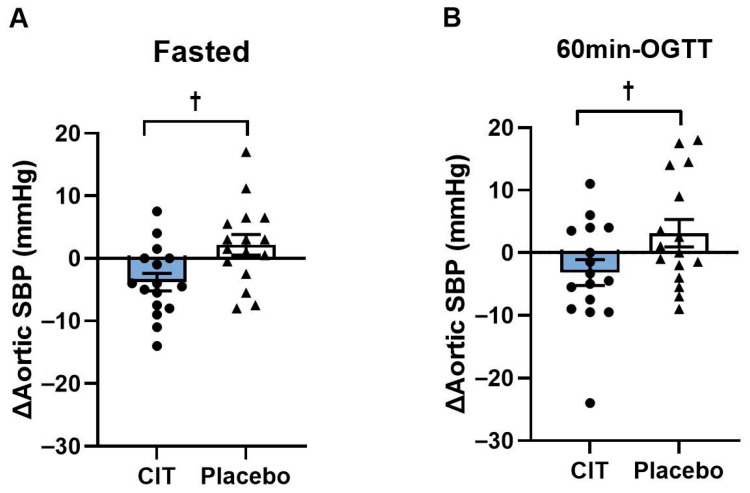
Changes (Δ) in aortic systolic blood pressure (SBP) from pre to post (**A**) in the fasted state and (**B**) at 60 min of oral glucose tolerance test (60 min-OGTT) in L-Citrulline (CIT) and placebo groups. ^†^ *p* < 0.05 vs. placebo.

There were no baseline differences in BP and heart rate between groups (all *p* > 0.05, Table 5). Significant group-by-time interactions for brachial SBP and PP, and aortic SBP and PP were found in the fasted state (all *p* < 0.05, Table 5) and for aortic SBP and MAP in the hyperglycemic state (all *p* < 0.05, Table 5). Brachial SBP and PP, and aortic SBP were reduced after CIT, whereas there were no changes in the placebo group (all *p* < 0.05, Table 5). Aortic SBP decreased in the fasted state (CIT: Δ−4 ± 1 mmHg vs. Placebo: Δ2 ± 2 mmHg, *p* < 0.05) and at 60 min-OGTT (CIT: Δ−3 ± 2 mmHg vs. Placebo: Δ3 ± 2 mmHg, *p* < 0.05) after CIT compared to placebo (Figure 5).

## 4. Discussion

This study investigated the effects of 4-week CIT supplementation on vascular function and glycemia in the fasted and acute hyperglycemic states in patients with T2DM. The main findings of this study are that 4 weeks of CIT supplementation greatly improved brachial artery FMD, blood glucose levels, HbA1c, faPWV, and brachial and aortic SBP in the fasted state. In addition, at 60 min of acute hyperglycemia, CIT supplementation increased FMD and decreased blood glucose levels, faPWV, and SBP (brachial and aortic). Taken together, CIT supplementation for 4 weeks improves brachial artery endothelial function, peripheral arterial stiffness, brachial and aortic SBP, and glycemic control in the fasted and hyperglycemic conditions in middle-aged and older adults with T2DM. These findings suggest that 4 weeks of CIT supplementation may be a potential strategy to diminish the adverse effects of chronic and acute hyperglycemia on arterial function and glucose regulation in patients with T2DM.

Endothelial dysfunction is strongly associated with vascular complications and CVD risk in T2DM [30]. Reduced NO bioavailability secondary to insulin resistance and chronic hyperglycemia is the main cause of endothelial dysfunction in T2DM [2,3,18]. In this study, 4 weeks of CIT supplementation increased brachial FMD by 1.3% in individuals with T2DM, indicating improved endothelial function. Considering a 2 to 3 times higher risk of CVD mortality among individuals with T2DM than individuals without T2DM [31], this improvement in FMD has a clinical significance because it is potentially associated to decrease future CVD events by approximately 17% [32]. Consistent with our findings, long-term (4–8 weeks) and short-term (7 days) CIT supplementation improved FMD in postmenopausal women [22] and patients with heart failure [23]. The improvement in FMD after CIT supplementation may be through increased circulating ARG levels [22], the substrate for endothelial NO synthesis. Another potential mechanism for improved FMD by CIT may be through inhibition of arginase activity. Arginase is an enzyme that metabolizes ARG to L-ornithine, reducing ARG availability to eNOS [33]. Shatanawi et al. demonstrated decreased arginase activity and increased plasma NO levels in people with T2DM after CIT (2 g/day) supplementation for 4 weeks, suggesting that CIT supplementation may increase endothelial NO production by arginase inhibition in T2DM [17]. However, the previous study did not examine endothelial function. Decreased arginase activity may result from reduced reactive oxygen species (ROS) production due to enhanced ARG availability leading to endothelial NO synthesis [18,34]. We recently reported an increase in plasma ARG levels in the same cohort of T2D participants following CIT supplementation [35]. Taken together, our findings suggest that CIT supplementation is effective to improve peripheral artery endothelial function in patients with T2DM.

We found that 4 weeks of CIT supplementation reduced faPWV (peripheral arterial stiffness) in the fasted state, but did not affect cfPWV (aortic stiffness). Similarly, Ochiai et al. reported a significant decrease in baPWV (systemic arterial stiffness) together with increased plasma ARG and NO levels in middle-aged men after 7 days of CIT (5.6 g/day) supplementation [25]. Figueroa et al. also showed that 8 weeks of CIT (6 g/day) supplementation reduced baPWV and faPWV, but not cfPWV in obese postmenopausal women [24]. It is possible that the reduction in faPWV after 4 weeks of CIT may be due to a functional (i.e., vasodilatory capacity), but not a structural improvement. On the other hand, the absence of an improvement in cfPWV (aortic) may be mostly due to different characteristics of central and peripheral arteries [36]. The central arteries become stiffer with aging and diabetes [5,37] by losing elastin and accumulating collagen, while the peripheral arteries that contain more smooth muscle cells are not similarly affected by aging [36,37]. Since cfPWV is an indicator of aortic stiffness reflecting the long-term structural remodeling of large elastic arteries [38], it seems that 4 weeks of CIT is insufficient to reverse the age- and disease-related structural characteristics [39] in our middle-aged and older adults with T2DM. Taken together, 4 weeks of CIT supplementation can reduce peripheral arterial stiffness, but a longer duration of supplementation might be needed to reduce aortic stiffness. Our finding of a reduction in faPWV has a clinical significance since leg arterial stiffness (faPWV or baPWV) is associated with systolic hypertension [36], abdominal obesity, insulin resistance [40], and muscle mass decline [41]. Moreover, increased peripheral arterial stiffness can contribute to sarcopenia by reducing blood supply to the legs in T2DM [42,43]; therefore, reduced faPWV after CIT supplementation may be helpful to attenuate or reverse sarcopenia in T2DM.

We also found that CIT supplementation decreased both brachial and aortic SBP by 4 mmHg in the fasted state in patients with T2DM. In agreement with our findings, previous studies have observed that 8 weeks of CIT supplementation reduced resting brachial and aortic SBP in obese postmenopausal women with elevated BP and hypertension [28]. These reductions in BP may be attributed to improved endothelial function and peripheral arterial stiffness, as discussed above. Another possibility may be related to reduced sympathetic tone. Obesity increases systemic sympathetic activity, causing vasoconstriction and increased peripheral vascular resistance, which drives the development of hypertension [44]. Although we did not measure sympathetic nerve activity, our data showed a significant reduction in SBP with decreased waist circumference (abdominal obesity) and arterial stiffness after 4 weeks of CIT supplementation in patients with T2DM. The decrease in SBP has a potential clinical benefit since a 5 mmHg reduction is associated with a 10% reduction in CVD incidence [45].

Four weeks of CIT supplementation decreased fasting HbA1C and blood glucose levels. Previous studies have shown that 8 weeks of CIT (3 g/day) supplementation decreased fasting blood glucose, HbA1c, and insulin resistance (HOMA-IR) in T2DM [19,21]. We observed that CIT reduced HbA1c by 0.3–1.1% in 7 participants. This is clinically important since intensive and long-term glycemic control with medical treatments have reduced HbA1c by 1% with an associated reduced risk of microvascular complications in older adults with T2D [46]. Improved insulin sensitivity can stimulate insulin-mediated NO production in the endothelium [47], leading to increases in blood flow and delivery of insulin and glucose primarily to skeletal muscles [48], increasing glucose uptake into the cells [49]. Although we did not observe a significant change in HOMA-IR, our findings suggest that CIT supplementation is beneficial to regulate blood glucose levels by improving insulin-dependent glucose uptake in T2DM through enhanced peripheral arterial dilation. Considering that chronic hyperglycemia is a characteristic of T2DM and the main cause of vascular dysfunction [2], our findings have clinical importance as a potential strategy for patients with T2DM to reduce CVD risk.

For the management of T2D, postprandial glucose has been commonly targeted at < 140 mg/dL at 2 h after the start of meals [50]. Both the American Diabetes Association and the International Diabetes Federation guidelines suggested focusing on postprandial glucose after 1 h post ingestion [51,52]. Several studies have demonstrated that 1-h postprandial glucose is a better predictor for the of CVD and mortality [27,53]. This increased CVD risk may be related to the lowest FMD value, indicative of acute exacerbation of endothelial dysfunction, observed at 1-h of OGTT in patients with T2D [10]. We found an attenuated increase in blood glucose levels during OGTT together with improved FMD after 4 weeks of CIT supplementation. In contrast, Vincellette et al., reported no improvements in blood glucose or FMD% during OGTT after 2 weeks of watermelon supplementation (195 and 795 mg of ARG and CIT per day, respectively) in healthy young adults [26]. This discrepancy may be due to short duration intervention, healthy young population, and not enough CIT content in watermelon juice. Our findings indicate that 4-week CIT supplementation is effective in reversing the adverse influence of acute hyperglycemia on vascular function in patients with T2DM. This may be due to an improved glucose uptake into skeletal muscle [49] reduces blood glucose concentration after glucose ingestion [54]. Another possibility is a decreased hepatic glucose release to the circulation [55]. In female rats with T2DM, CIT administration decreased fasting glucose, improved glucose tolerance, and reduced gluconeogenesis [55], suggesting that CIT stimulates insulin secretion and increases NO production, which can inhibit gluconeogenesis in hepatocytes [56]. The lower increase in blood glucose attenuates ROS production [3], reduces arginase activity and increases NO production [3,18,47].

There are a few limitations in this study. First, we included three patients with T2DM on insulin injection therapy and/or anti-hypertensive medications. However, they were stable and well-controlled (treating glucose and/or BP > 3 months). A previous study also included patients with T2DM on insulin and/or anti-hypertensive medications and examined vascular function after NO-enhancing supplementation [57]. In a sub-analysis separating these 3 participants who were taking on insulin therapy, the improvement in FMD after CIT supplementation was not different between people with T2DM on insulin therapy and those only on oral-hypoglycemic medications. Second, we examined vascular function using non-invasive methods in middle-aged and older men and women with T2DM, but the study was not powered to detect sex differences. Future studies are needed to investigate the long-term effect of CIT supplementation on vascular function, visceral fat mass, and lean mass considering sex differences in individuals with T2DM. Third, our findings cannot be generalized to the T2D population since young adults were not included. We focused on middle-aged and older adults because they have higher comorbidity rates, including obesity-hypertension and sarcopenia, compared to young individuals with T2D [58]. Lastly, we did not measure visceral adipose tissue and blood markers such as NO metabolites, advanced glycation end products, adipokines (e.g., adiponectin, leptin, visfatin), and oxidative stress markers, which would provide further insight into potential mechanisms behind our findings. However, we assessed NO-mediated endothelial function using brachial FMD [59] and found significant improvements with CIT supplementation. In addition, previous studies have demonstrated the effectiveness of CIT supplementation on improving insulin resistance, arginase activity, oxidative stress, and inflammation in T2DM [17,19,20,21].

## 5. Conclusions

In conclusion, 4 weeks of CIT supplementation improved brachial artery endothelial function, leg arterial stiffness, aortic SBP, and blood glucose levels in the fasted state. Moreover, CIT supplementation attenuated the increase in blood glucose levels with improvements in peripheral artery endothelial function and stiffness, and in aortic SBP during OGTT. Therefore, our findings suggest that CIT supplementation improves vascular function in the fasted and acute hyperglycemic states and may be an effective therapeutic strategy to improve vascular function in middle-aged and older adults with T2DM.

## Figures and Tables

**Table 1:** Participant characteristics at baseline.

	Value (n = 16)
Age (years)	62 ± 6
Sex (male/female)	7/9
Diabetes duration (years)	8 ± 5
Height (m)	1.66 ± 0.12
Weight (kg)	93.0 ± 18.7
BMI (kg/m^2^)	33.4 ± 4.9
Waist circumference (cm)	114.4 ± 11.6
Brachial SBP (mmHg)	130 ± 12
Fasting blood glucose (mg/dl)	139 ± 29
HbA1C (mmol/mol)	44 ± 8
HbA1C (%)	6.3 ± 0.8

Values are mean ± standard deviation. Abbreviations: BMI, body mass index; SBP, systolic blood pressure; HbA1C, hemoglobin A1C.

**Table 2:** Anthropometrics and glucose at baseline (pre) and 4 weeks (post).

	CIT (n = 16)		Placebo (n = 16)		*p*
	Pre	Post	Pre	Post	
Height (m)	1.66 ± 0.12	-	1.66 ± 0.12	-	-
Weight (kg)	92.9 ± 18.2	92.4 ± 18.3	92.1 ± 18.5	92.5 ± 18.2	0.08
BMI (kg/m^2^)	33.4 ± 5.1	33.2 ± 4.9	33.1 ± 4.8	33.3 ± 4.9	<0.05
Waist circumference (cm)	114.3 ± 11.8	111.8 ± 12.4 *^,†^	113.2 ± 11.7	113.5 ± 11.6	<0.05
HbA1C (mmol/mol)	45 ± 9	42 ± 7 *^,†^	44 ± 9	46 ± 9	<0.01
HbA1C (%)	6.3 ± 0.8	6.0 ± 0.7 *^,†^	6.2 ± 0.8	6.4 ± 0.8	<0.01
Glucose (mg/dL)	157 ± 29	144 ± 30 *	140 ± 26	151 ± 33	<0.01
Insulin (µIU/mL)	27 ± 18	20 ± 10 *	23 ± 9	28 ± 12	<0.05
HOMA-IR	8.9 ± 4.9	7.6 ± 4.0	8.7 ± 4.0	9.8 ± 4.6	0.16

Values are mean ± standard deviation. Abbreviations: CIT, L-citrulline; BMI, body mass index; HbA1C, hemoglobin A1C; HOMA-IR, homeostatic model assessment for insulin resistance. *p*-values are the group-by-time interaction from two-way repeated measures ANOVA. * *p* < 0.05 vs. pre; ^†^ *p* < 0.05 vs. placebo.

**Table 3:** Blood glucose levels in the fasted and acute hyperglycemic states at baseline (pre) and 4 weeks (post).

	CIT (n = 16)		Placebo (n = 16)		*p*
	Pre	Post	Pre	Post	
Glucose (mg/dL)					
fasted	157 ± 29	144 ± 30 *	140 ± 26	151 ± 33	<0.01
60 min-OGTT	267 ± 59	236 ± 49 **^,†^	254 ± 63	274 ± 57 *	<0.01

Values are mean ± standard deviation. Abbreviations: CIT, L-citrulline; 60 min-OGTT, 60 min during oral glucose tolerance test. *p*-values are the group-by-time interaction from three-way repeated measures ANOVA. * *p* < 0.05 vs. pre; ** *p* < 0.01 vs. pre; ^†^ *p* < 0.05 vs. placebo.

**Table 4:** Macrovascular endothelial function in the fasted and acute hyperglycemic states at baseline (pre) and 4 weeks (post).

	CIT (n = 16)		Placebo (n = 16)		*p*
	Pre	Post	Pre	Post	
Baseline diameter (mm)					
fasted	3.71 ± 0.50	3.80 ± 0.55	3.79 ± 0.59	3.78 ± 0.60	0.29
60 min-OGTT	3.72 ± 0.59	3.73 ± 0.57	3.78 ± 0.64	3.72 ± 0.61	0.60
Peak diameter (mm)					
fasted	3.88 ± 0.52	4.02 ± 0.54 *	3.96 ± 0.60	3.98 ± 0.58	0.13
60 min-OGTT	3.82 ± 0.60	3.87 ± 0.58	3.88 ± 0.65	3.82 ± 0.62	0.26
Brachial FMD (%)					
fasted	4.3 ± 1.4	5.6 ± 1.6 **^,‡^	4.7 ± 1.4	4.2 ± 1.2 **	<0.01
60 min-OGTT	2.6 ± 1.1	3.9 ± 1.2 **^,‡^	2.7 ± 1.1	2.3 ± 1.3 *	<0.01
Baseline shear rate (sec^−1^)					
fasted	130 ± 69	184 ± 219	127 ± 38	240 ± 254	0.46
60 min-OGTT	125 ± 50	176 ± 152	130 ± 51	154 ± 155	0.62
Peak shear rate (sec^−1^)					
fasted	958 ± 304	1015 ± 370	988 ± 295	1123 ± 540	0.53
60 min-OGTT	987 ± 342	1079 ± 442	943 ± 304	926 ± 323	0.47

Values are mean ± standard deviation. Abbreviations: CIT, L-citrulline; 60 min-OGTT, 60 min during oral glucose tolerance test; FMD, flow-mediated dilation. *p*-values are the group-by-time interaction from three-way repeated measures ANOVA. * *p* < 0.05 vs. pre; ** *p* < 0.01 vs. pre; ^‡^ *p* < 0.01 vs. placebo.

**Table 5:** Arterial stiffness and brachial and aortic blood pressure in the fasted and acute hyperglycemic states at baseline (pre) and 4 weeks (post).

	CIT (n = 16)		Placebo (n = 16)		*p*
	Pre	Post	Pre	Post	
cfPWV (m/s)					
fasted	8.5 ± 1.0	8.3 ±1.3	8.0 ± 1.1	7.9 ± 1.1	0.37
60 min-OGTT	8.5 ± 1.0	8.3 ± 1.4	8.3 ± 0.9	8.2 ± 0.8	0.32
faPWV (m/s)					
fasted	9.1 ± 0.9	8.5 ± 1.0 **^,†^	8.7 ± 0.8	9.3 ± 0.6 **	<0.01
60 min-OGTT	9.6 ± 0.7	8.8 ± 0.8 **^,‡^	9.2 ± 0.7	9.9 ± 0.8 **	<0.01
Brachial SBP (mmHg)					
fasted	143 ± 11	139 ± 11 **	137 ± 13	139 ± 13	<0.01
60 min-OGTT	147 ± 15	143 ± 13	142 ± 14	145 ± 15	0.05
Brachial DBP (mmHg)					
fasted	85 ± 9	84 ± 9	84 ± 10	84 ± 10	0.46
60 min-OGTT	85 ± 11	84 ± 11	84 ± 11	87 ± 13	0.14
Brachial MAP (mmHg)					
fasted	104 ± 9	102 ± 9 *	102 ± 10	102 ± 11	0.08
60 min-OGTT	105 ± 12	104 ± 11	103 ± 11	106 ± 13	0.06
Brachial PP (mmHg)					
fasted	46 ± 7	55 ± 7 **	42 ± 6	55 ± 8 **	<0.05
60 min-OGTT	62 ± 7	60 ± 8	58 ± 8	57 ± 10	0.40
Aortic SBP (mmHg)					
fasted	132 ± 11	128 ± 11 *	126 ± 12	129 ± 12	<0.05
60 min-OGTT	134 ± 15	130 ± 13 *	129 ± 13	132 ± 14	<0.05
Aortic DBP (mmHg)					
fasted	86 ± 9	84 ± 8	85 ± 10	85 ± 10	0.51
60 min-OGTT	85 ± 11	85 ± 11	85 ± 10	89 ± 14 *	0.08
Aortic MAP (mmHg)					
fasted	105 ± 10	102 ± 9	102 ± 11	103 ± 11	0.06
60 min-OGTT	105 ± 13	104 ± 13	103 ± 12	107 ± 14 *	<0.05
Aortic PP (mmHg)					
fasted	46 ± 7	44 ± 5	42 ± 6	44 ± 6 *	<0.01
60 min-OGTT	48 ± 6	46 ± 6	44 ± 6	43 ± 8	0.42
Heart Rate (bpm)					
fasted	68 ± 9	69 ± 8	71 ± 11	71 ± 10	0.38
60 min-OGTT	72 ± 10	73 ± 9	75 ± 11	75 ± 11	0.75

Values are mean ± standard deviation. Abbreviations: CIT, L-citrulline; 60 min-OGTT, 60 min during oral glucose tolerance test; cfPWV, carotid-femoral pulse wave velocity; faPWV, femoral-ankle pulse wave velocity; SBP, systolic blood pressure; DBP, diastolic blood pressure; MAP, mean arterial pressure; PP, pulse pressure. *p*-values are the group-by-time interaction from three-way repeated measures ANOVA. * *p* < 0.05 vs. pre; ** *p* < 0.01 vs. pre; ^†^ *p* < 0.05 vs. placebo; ^‡^ *p* < 0.01 vs. placebo.

## Data Availability

The raw data supporting the conclusions of this article will be made available by the authors on request.

## Supplementary Materials

The following supporting information can be downloaded at: https://www.mdpi.com/article/10.3390/nu17233739/s1, Table S1: Participant medications.
