# Supplementary material for: Effects of L-Citrulline Supplementation on Endothelial Function, Arterial Stiffness, and Blood Glucose Level in the Fasted and Acute Hyperglycemic States in Middle-Aged and Older Adults with Type 2 Diabetes
Source: Nutrients. 2025 Nov 28;17(23):3739. doi: 10.3390/nu17233739 (PMC12693867; doi:10.3390/nu17233739)
Supplement: Supplementary file 1 [file nutrients-17-03739-s001.zip › nutrients-3982304-supplementary.pdf]

Table S1. Participant medications.

**Hypoglycemic treatment, n (%)**	
Metformin	13 (81)
Sulfonylureas	5 (31)
SGLT2 inhibitor	1 (6)
GLP-1 receptor agonist	4 (25)
Thiazolidinedione	1 (6)
Insulin	3 (19)
**Anti-hypertensive medications, n (%)**	
ACE inhibitor	4 (25)
ARB	5 (31)
Calcium channel blocker	2 (13)
Diuretic	4 (25)
Statin	9 (56)
**Hormone replacement therapy, n (%)**	
Estradiol	1 (6)
Progesterone	1 (6)
Levothyroxine	1 (6)

Values are number of subjects (%). Abbreviations: SGLT2, sodium-glucose co-transporter 2; GLP-1, glucagon-like peptide 1; ACE, angiotensin-converting enzyme; ARB, angiotensin II receptor blocker.
